# Editorial Comment: Best practices in near-infrared fluorescence imaging with indocyanine green (NIRF/ICG)-guided robotic urologic surgery: a systematic review-based expert consensus

**DOI:** 10.1590/S1677-5538.IBJU.2020.02.09

**Published:** 2020-01-10

**Authors:** Eliney F. Faria

**Affiliations:** 1 Serviço de Urologia Hospital de Amor BarretosSP Brasil Serviço de Urologia, Hospital de Amor de Barretos, SP, Brasil;; 2 Serviço de Urologia Hospital Felicio Rocho Belo HorizonteMG Brasil Serviço de Urologia, Hospital Felicio Rocho – Belo Horizonte, MG, Brasil

Cacciamani GE ^1^, Shakir A ^2^, Tafuri A ^2,3^, Gill K ^2^, Han J ^2^, Ahmadi N ^2,4^, Hueber PA ^2^, Gallucci M ^5^, Simone G ^5^, Campi R ^6,7^, Vignolini G ^6,7^, Huang WC ^8^, Taylor J ^8^, Becher E ^8^, Van Leeuwen FWB ^9,10,11^, Van Der Poel HG ^9^, Velet LP ^12^, Hemal AK ^12^, Breda A ^13^, Autorino R ^14^, Sotelo R ^2^, Aron M ^2^, Desai MM ^2^, De Castro Abreu AL ^2^

^1^ USC Institute of Urology and Catherine and Joseph Aresty Department of Urology, Keck School of Medicine, University of Southern California, Los Angeles, CA, USA; ^2^ USC Institute of Urology and Catherine and Joseph Aresty Department of Urology, Keck School of Medicine, University of Southern California, Los Angeles, CA, USA; ^3^ Department of Urology, University of Verona, Verona, Italy; ^4^ Department of Uro-Oncology, Chris O’Brien Lifehouse, Camperdown, NSW, Australia; ^5^ Department of Urology, “Regina Elena” National Cancer Institute, Rome, Italy; ^6^ Department of Urologic Robotic Surgery and Renal Transplantation, Careggi Hospital, University of Florence, Florence, Italy; ^7^ Department of Experimental and Clinical Medicine, University of Florence, Florence, Italy; ^8^ Division of Urologic Oncology, Department of Urology, NYU Langone Health, New York, USA; ^9^ Department of Urology, Antoni van Leeuwenhoek Hospital, Netherlands Cancer Institute, Amsterdam, The Netherlands; ^10^ Interventional Molecular Imaging Laboratory, Leiden University Medical center, Leiden, The Netherlands; ^11^ Orsi Academy, Melle, Belgium; ^12^ Department of Urology, Wake Forest University, Winston-Salem, NC, USA; ^13^ Fundació Puigvert, Department of Urology, Autonomous University of Barcelona, Barcelona, Spain; ^14^ Division of Urology, Department of Surgery, VCU Health, Richmond, VA, USA

World J Urol. 2019; 8. [Epub ahead of print]

**DOI:** 10.1007/s00345-019-02870-z | **ACCESS:** 10.1007/s00345-019-02870-z

## COMMENT

In this very nice paper Dr Cacciamani e other experts explained about use of indocyanine green (ICG) to allow visualization of both the vasculature and contours of anatomic structures (1). The use of near-infrared fluorescence (NIRF) technology with ICG has been explored in several surgical specialties (2). It is able to provide an enhanced anatomical view of the surgical field with potentially improved perioperative surgical outcomes (3, 4). They highlight the potential uses of NIRF with ICG for robotic urologic surgery integrating with Firefly® technology in the Da Vinci Surgical platform and systematically (guidelines set out by PRISMA) investigated the impact of the this technology in robotic urologic surgery (5). Interestingly they generated several QRcodes to link a video-clip to readers. They reported in a review that NIRF/ICG technology has emerged as an interesting tool improving the identification of anatomical landmarks for oncological and nononcological procedures. This approach facilitate challenging reconstructive and oncologic robotic procedures. The NIRF with ICG can be used in robotic partial nephrectomy (RPN) mainly to localize small branches and perform selective clamping. This approach is useful to discern between pathological and normal renal tissue (6). In robotic-assisted radical prostatectomy (RARP) it can be used for to visualize the arteries of neurovascular bundle or helping identification of regional lymph nodules. It can better-assist in understanding lymphatic drainage improving diagnostic findings (7, 8). Furthermore, they described the use of this technology during robotic surgery for different types of adrenal pathologies helping in adrenal-sparing surgery (9). They showed also encouraging studies for use in robotic inguinal lymphadenectomy (10). In robotic radical cystectomy, the use of NIRF/ICG-guided can be helpful in identification of sentinel nodes and assessing the vascularity of bowel avoiding mesenteric arcades during intracorporeal deviation. The authors also cited the use during robotic ureteral re-implantation/reconstruction in the evaluation of the vascularity of the ureteral margins and during kidney transplant. However the paper described despite NIRF in urology are promising, the level of evidence is low. Further investigations are needed to improve the understanding on the technology.
